# Depositing a Histone That Protects Active Chromosomal Regions from Silencing

**DOI:** 10.1371/journal.pbio.0020136

**Published:** 2004-03-23

When James Watson and Francis Crick reported the structure of DNA in 1953, the mechanism of inheritance was instantly apparent. The complementary pairing of the DNA bases in the double helix, the pair famously wrote, “immediately suggests a possible copying mechanism for the genetic material.” The structure helped explain one of the central problems of modern biology: how does genetic material get faithfully replicated and then passed on from generation to generation? It was long thought that DNA is the only unit of inheritance.[Fig pbio-0020136-g001]

**Figure pbio-0020136-g001:**
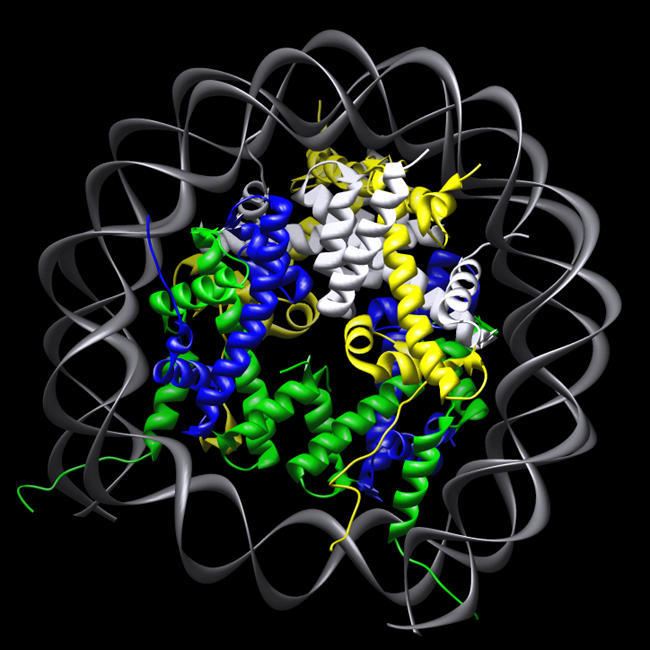
Nucleosome containing H2A.Z

Since then, it's become clear that molecules of DNA are packaged into highly organized superstructures that themselves are inherited. These structures play a significant role in the regulation of genes by preventing or facilitating protein–DNA interactions. In the eukaryotic cell (a cell with a nucleus), DNA exists as long threadlike molecules—a typical human cell contains some 6.5 feet (2 meters) of DNA—that associate with a variety of proteins to form a network called chromatin. Genomic DNA wraps around specialized DNA-packing proteins called histones to form nucleosomes, which condense chromatin into chromosomes and thereby influence chromosome behavior. Chromosomes are in turn packaged in increasingly higher levels of organization, with some parts being dispersed and others condensed. The most condensed region is called heterochromatin, or silent chromatin. Gene expression is largely silent in these regions, since the proteins required for transcription can't access DNA to transcribe genes when chromosomes are so tightly packed. Other regions of chromosomes exist in an extended state, called euchromatin. This is the most genetically active state; with genes exposed, transcription can easily occur.

As chromatin shifts between these states, it influences gene expression, largely through the interactions of histones and large protein complexes that together assemble, remodel, and modify chromatin. Since proper cell function depends largely on activating the right gene at the right time, mechanisms have evolved that protect active genes from the intrusions of silencing structures like heterochromatin. Both euchromatin and heterochromatin respond to mechanisms that resist encroachments of the opposite state. One mechanism involves replacing “canonical” (that is, archetypal) histones with a histone variant. Previous work on yeast from Hiten Madhani and colleagues had shown that one histone variant, called H2A.Z, is found specifically in euchromatin and prevents silent chromatin from spreading into adjacent euchromatic regions. While researchers have characterized some of the mechanisms that deposit canonical histones onto euchromatin, they knew little about the mechanisms that deposit variant histones. In this issue of *PLoS Biology,* Jasper Rine, Hiten Madhani, and colleagues identify and characterize the function of a protein complex that helps deposit the variant H2A.Z onto euchromatin in yeast.

To investigate which proteins help direct H2A.Z to specific chromosomal locations, the authors isolated H2A.Z, along with whatever proteins were associated with it, from yeast cell extracts. They determined that 15 proteins were true binding partners of H2A.Z and that 13 of them form a complex called SWR1-Com. The largest subunit of this complex, called Swr1p, belongs to a well-known family of adenosine triphosphate (ATP)-dependent chromatin remodeling enzymes (they use the energy of ATP to power remodeling) that provide access to DNA in chromatin. Rine, Madhani, and colleagues show that protein subunits of SWR1-Com associate specifically with the histone variant H2A.Z. By comparing the gene expression profiles of yeast mutants lacking the H2A.Z-encoding gene with mutants lacking the Swr1p-encoding gene, the authors show that H2A.Z depends on the SWR1-Com protein complex to function. Most importantly, they show that SWR1-Com is required in living cells to deposit H2A.Z onto euchromatin. Interestingly, the authors note, SWR1-Com shares subunits with a histone-acetylating enzyme involved in the regulation of transcription (called the NuA4 histone acetyltransferase) and with another chromatin remodeler, which suggests that biochemical modifications of the subunits on histone “tails” may play a role in replacing H2A with H2A.Z.

This histone–protein complex, the authors conclude, represents a chromatin remodeling machine with a novel function, revealing a new role for Swr1p-type enzymes and a novel mechanism of genome regulation. By preventing the spread of silent chromatin into transcriptionally active chromosomal regions—the result of the interaction described here—this mechanism allows the cell's gene expression program to operate with precision and on schedule. Since chromosomes can be inherited by daughter cells in this active state, such mechanisms ensure that gene expression programs essential for ongoing fundamental processes like embryogenesis and cellular differentiation proceed without interference.

